# Synergistic Interactions between the Hypomethylating Agent Thio-Deoxycytidine and Venetoclax in Myelodysplastic Syndrome Cells

**DOI:** 10.3390/hematolrep15010010

**Published:** 2023-02-02

**Authors:** Xiaoyan Hu, Lin Li, Jewel Nkwocha, Kanika Sharma, Liang Zhou, Steven Grant

**Affiliations:** 1 Division of Hematology/Oncology, Department of Medicine, Virginia Commonwealth University, Richmond, VA 23298, USA; 2 Massey Cancer Center, Virginia Commonwealth University, Richmond, VA 23298, USA

**Keywords:** T-dCyd, ABT-199/venetoclax, myelodysplastic syndrome, ROS

## Abstract

Interactions between the novel hypomethylating agent (HMA) thio-deoxycytidine (T-dCyd) and the BCL-2 antagonist ABT-199 (venetoclax) have been examined in human myelodysplastic syndrome (MDS) cells. The cells were exposed to agents alone or in combination, after which apoptosis was assessed, and a Western blot analysis was performed. Co-administration of T-dCyd and ABT-199 was associated with the down-regulation of DNA methyltransferase 1 (DNMT1) and synergistic interactions documented by a Median Dose Effect analysis in multiple MDS-derived lines (e.g., MOLM-13, SKM-1, and F-36P). Inducible BCL-2 knock-down significantly increased T-dCyd’s lethality in MOLM-13 cells. Similar interactions were observed in the primary MDS cells, but not in the normal cord blood CD34^+^ cells. Enhanced killing by the T-dCyd/ABT-199 regimen was associated with increased reactive oxygen species (ROS) generation and the down-regulation of the anti-oxidant proteins Nrf2 and HO-1, as well as BCL-2. Moreover, ROS scavengers (e.g., NAC) reduced lethality. Collectively, these data suggest that combining T-dCyd with ABT-199 kills MDS cells through an ROS-dependent mechanism, and we argue that this strategy warrants consideration in MDS therapy.

## 1. Introduction

Myelodysplastic syndrome (MDS) represents a group of malignant disorders involving myeloid hematopoietic stem cells, which generally culminates in bone marrow failure and/or transformation to acute myelogenous leukemia (AML) [1]. Among the approved treatments for MDS, attention has focused on hypomethylating agents such as 5-azacytidine and decitabine, which are believed to act by inducing the degradation of DNA methyltransferase 1 (DNMT1), leading to the demethylation of CpG islands and the enhanced expression of genes required for normal myeloid hematopoiesis [2]. Recently, it has been shown that the co-administration of HMAs with the BCL-2 antagonist venetoclax (ABT-199) results in a marked increase in therapeutic activity, and this strategy has been approved for older patients with AML who are not candidates for more aggressive approaches [3]. The mechanisms underlying HMA/ABT-199 synergism in AML may include the disruption of the electron transport chain, particularly Complex IV, oxidative injury, and the eradication of leukemia stem cells [4,5]. Although they are preliminary, there have been suggestions that the HMA/ABT-199 strategy might also offer promise for patients with MDS [6].

Thio-deoxycytidine (T-dCyd) and related agents (e.g., aza-T-dCyd) represent novel HMAs that have the advantage of being administered orally [7]. In pre-clinical studies, T-dCyd has been shown to induce potent DNMT1 down-regulation and to exhibit activity in solid tumor models [8]. Based on these considerations, trials of T-dCyd in patients with MDS or solid tumor malignancies have been initiated at the NCI (Clin Trials.GOV_NCT02423057).

Currently, not a lot is known from a pre-clinical standpoint about the interactions between HMAs and ABT-199 in MDS models, and no information is available concerning HMAs such as T-dCyd. In addition, it is unknown whether the mechanisms underlying the synergistic interactions between HMAs and ABT-199 can be extrapolated to MDS systems. The purpose of this study was to characterize T-dCyd/ABT-199 interactions in various MDS-derived cells and to shed light on the potential mechanisms responsible for synergism in such systems.

## 2. Material and Methods

### 2.1. Cell Lines and Reagents

Human MDS-derived cell lines MOLM-13, SKM-1, F-36P, and AML cell lines U937 and MV-4-11 were used in this study. For all of the experiments, the cells were maintained in RPMI-1640 supplemented with 10% fetal bovine serum and penicillin-streptomycin. All of the experiments utilized logarithmically grown cells (3–4 × 10^5^ cells/mL). MycoAlert (Lonza, Allendale, NJ, USA) assays were performed, demonstrating that all of the cell lines were free of mycoplasma contamination. Inducible MOLM-13 BCL-2 knock-down cells were obtained as previously described [9].

We obtained 5-Aza-T-dCyd and T-dCyd from the Developmental Therapeutics Program, Division of Cancer Treatment and Diagnosis, National Cancer Institute (Rockville, MD, USA), and the Frederick National Laboratory, Frederick, MD, USA. ABT-199 was purchased from ChemieTek (Indianapolis, IN, USA). N-acetyl-L-cysteine (NAC) was purchased from Sigma-Aldrich (St. Louis, MO, USA). All of the drugs were dissolved in DMSO and reconstituted to 10 mM. The final DMSO concentrations did not exceed 0.1%. Aliquots of the drug were stored at −80 °C until use.

### 2.2. Analysis of Cell Death

Cell death was evaluated by flow cytometry and assessed by double staining with Annexin V-FITC/Propidium Iodide (PI), as it has been before [10].

### 2.3. Measurement of ROS

For the determination of the level of ROS, nontreated or treated cells were incubated with 10 uM 2′,7-dichlorodihydrofluorescein diacetate dye (DCFH-DA; Sigma-Aldrich) for the total ROS analysis or with 3 uM of MitoSOX Red (Life Technologies-Molecular Probes, Carlsbad, CA, USA) for the mitochondrial ROS analysis in culture media for 30 min at 37 °C. The cells were then washed with phosphate-buffered saline (PBS) and stained for the annexin-V/DAPI analysis using flow cytometry. Only the live cell population (annexin-V-/DAPI) was analyzed for intracellular and total ROS production. To determine the effect of N-acetyl-L-cysteine (NAC), an ROS scavenger, the cells were cultured with and without 2.5 mM of NAC for 2 h, following exposure to T-dCyd, venetoclax, or T-dCyd/venetoclax in combination for an additional 12 h.

### 2.4. Western Blot

The cells (3–5 × 10^6^) were treated with varying concentrations of T-dCyd or aza-T-dCyd (0.5–1 µM) or Venetoclax (2–50 nM), alone and combination. Whole-cell extracts were prepared from M-PERTM Mammalian Protein Extraction Reagent (Thermo Scientific, Rockford, IL, USA) and quantified using a Coomassie Protein Assay Reagent (Pierce ThermoFisher Scientific, Rockford, IL, USA). Equal amounts of protein (20 µg) were separated by SDS-PAGE and electro-transferred onto the nitrocellulose membrane. The blots were probed with primary antibodies against β-actin (#A2066, Sigma-Aldrich, St. Louis, MO, USA), anti-cleaved PARP (#9541, Cell Signaling, Danvers, MA, USA), anti-cleaved Caspase-3 (#9661S, Cell Signaling), anti-DNMT1 (ab134148, Abcam, Cambridge, UK), anti-BCL-2 (#4223S, Cell Signaling), anti-Nrf2 (sc-365949, Santa Cruz, CA, USA), anti-HO-1 (#5853S, Cell Signaling), and anti-P84 (ab487, Abcam).

### 2.5. Immunofluorescence

After the 24 h treatment, the primary patient mononuclear cells were incubated with Annexin V-FITC (#556419, BD Biosciences, Franklin Lakes, NJ, USA) and anti-human CD34 antibody (#343606, Biolegend, San Diego, CA, USA) for 20 min. The slides were then mounted using DAPI (4′,6-diamidino-2-phenylindole.) Fluoromount-G (Southern Biotech, Birmingham, AL, USA). Images were captured using an Olympus IX71 Inverted System Microscope with a DP73, 17MP color camera (Tokyo, Japan).

### 2.6. Isolation of Primary MDS Cells and Normal CD34^+^ Cells

Bone marrow or peripheral blood samples from the patients with MDS were obtained with written informed consent. Normal hematopoietic CD34^+^ cells were obtained with informed consent from human umbilical cord blood obtained from patients undergoing normal deliveries. These studies have been approved by the Institutional Review Board of the Virginia Commonwealth University Health Sciences Center. In the performed experiments, the mononuclear cells were isolated by Ficoll-Hypaque gradient separation as previously described [11], and they represent the entire unsorted population, and a gated CD34+ cell population was analyzed utilizing the FCM assay.

### 2.7. Statistical Analysis

The significance of differences between the experimental conditions was determined using the Student’s *t*-test for unpaired observations. *p*-values for such differences were designated as follows: * *p* < 0.05; ** *p* < 0.01; *** *p* < 0.001. The values represent the means ± SD for at least 3 independent experiments performed in triplicate. Analysis of synergism was performed by Median Dose Effect analysis using the software Calcusyn (Biosoft) [12].

## 3. Results

### 3.1. ABT-199/T-dCyd or aza-T-dCyd Synergism in MDS Cell Lines

**Figure 1:**
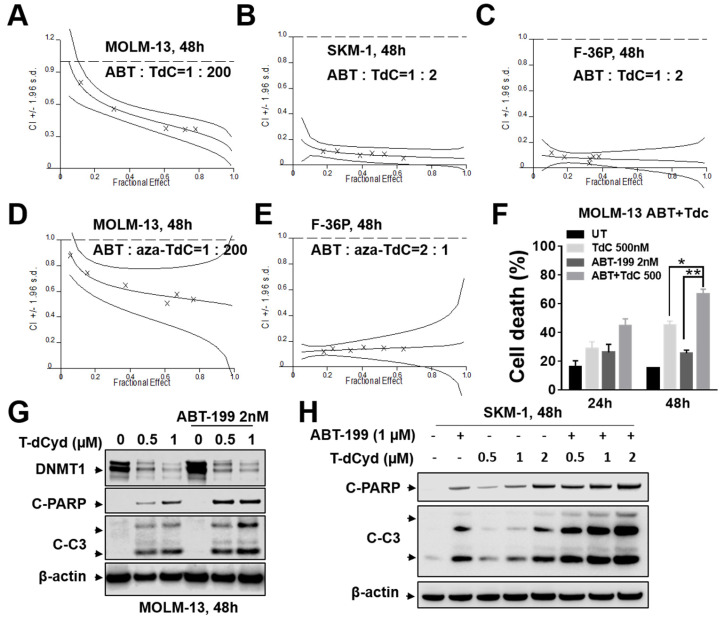
MDS–derived MOLM–13 and MDS SKM–1 and F–36P cells were exposed to varying concentrations of ABT–199 and T–dCyd or Aza–T–dCyd administered at a fixed ratio for 48 h, after which Median Dose Effect analysis was used to characterize concentration index (CI) values in relation to the fraction affected (FA). CI values < 1.0 denote synergistic interactions (**A**–**E**). (**F**) Cell death was monitored by Annexin V/PI staining and FCM. For *p* values, * = <0.05; ** = <0.01. (**G**,**H**) Cleaved PARP, caspase 3, and DNMT1 were detected by WB in MOLM–13 and SKM–1 cells exposed to T–dCyd ± ABT–199; β-actin controls are shown to document equivalent loading and transfer.

The MDS-derived MOLM-13 cells [13] were continuously exposed (48 h) to ABT-199 and T-dCyd at a concentration ratio of 1:200, after which apoptosis was determined by annexin/PI staining. The Median Dose Effect analysis yielded combination index values of less than 1.0, indicating synergistic interactions (Figure 1A). Similar results were obtained with two other MDS lines (SKM-1, F-36P; Figure 1B,C). Furthermore, synergism was also observed between ABT-199 and aza-T-dCyd in the MOML-13 and F-36P lines (Figure 1D,E). The significant increase in the apoptosis rate in the MOLM-13 cells with the combined treatment (both 24 and 48 h) with ABT-199/T-dCyd is shown in Figure 1F. The Western blot analysis of the MOLM-13 cells revealed that combined exposure to T-dCyd and ABT-199 resulted in a marked DNMT1 down-regulation and pronounced cleavage of PARP and caspase-3 (Figure 1G). A similar increase in PARP/caspse-3 cleavage was also observed in the SKM-1 cells (Figure 1H). These results indicate that thio-dCyd HMAs interact synergistically with ABT-199 in the MDS-derived cells.

### 3.2. ABT-199/T-dCyd or aza-T-dCyd Synergism in AML Cell Lines

Parallel studies were performed in AML lines not derived from MDS cells (e.g., MV4-11 and U937). In both the MV4-11 and U937 cells, both T-dCyd, as well as aza-T-dCyd, interacted synergistically with ABT-199 as per the Median Dose Effect analysis (C.I. values < 1.0) (Supplementary Figure S1A–D). The significant increase in cell death with the combined treatment at 48 h is shown in Supplementary Figure S1E,F. Finally, the combined treatment of MV4-11 (24 or 48 h) with ABT-199 and T-dCyd or aza-T-dCyd resulted in a marked DNMT1 down-regulation and a pronounced increase in PARP and caspase-3 cleavage (Supplementary Figure S1G). Together, these findings indicate that the synergistic interactions between T-dCyd or aza-T-dCyd and ABT-199 are similar in the MDS and non-MDS cell types.

### 3.3. BCL-2 Interruption Contributes to Enhanced T-dCyd Activity in MDS-Derived Cells

**Figure 2:**
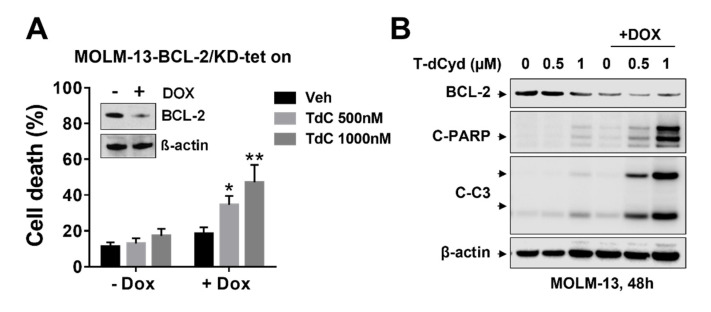
Dox–inducible TET–on MOLM–13 cells expressing BCL–2 shRNA were exposed to the indicated concentrations of T–dCyd in the presence or absence of doxycycline. Cell death was determined by annexin V/PI staining and FCM. Inset: expression of BCL–2 by WB with or without doxycycline. * (*p* < 0.05) and ** (*p* < 0.01) = significantly greater than values for cells without doxycycline (**A**); cleaved PARP and Caspase 3 were detected by WB (**B**).

To assess the functional significance of BCL-2 interruption, e.g., by ABT-199 in the -potential for T-dCyd susceptibility, a doxycycline-induced BCL-2 MOLM-13 knock-down cell line was established (Figure 2A). These cells exhibited a clear reduction of BCL-2 expression when they were cultured in the presence of doxycycline compared to cells cultured in the absence of doxycycline (Figure 2A, inset). Notably, the cells cultured with doxycycline were significantly more sensitive to T-dCyd-induced cell death compared to the cells cultured in the absence of doxycycline (Figure 2A). The Western blot analysis demonstrated that MOLM-13 cells cultured with doxycycline and exposed to T-dCyd exhibited a clear reduction of BCL-2 and a marked increase in PARP and caspase-3 cleavage compared to that of the controls (Figure 2B). These findings argue that interference with BCL-2 function plays a significant functional role in synergistic interaction between T-dCyd and ABT-199 in MDS-derived cells.

### 3.4. The T-dCyd/ABT-199 Regimen Is Active against Primary MDS, but Not Normal CD34^+^ Cells

**Figure 3:**
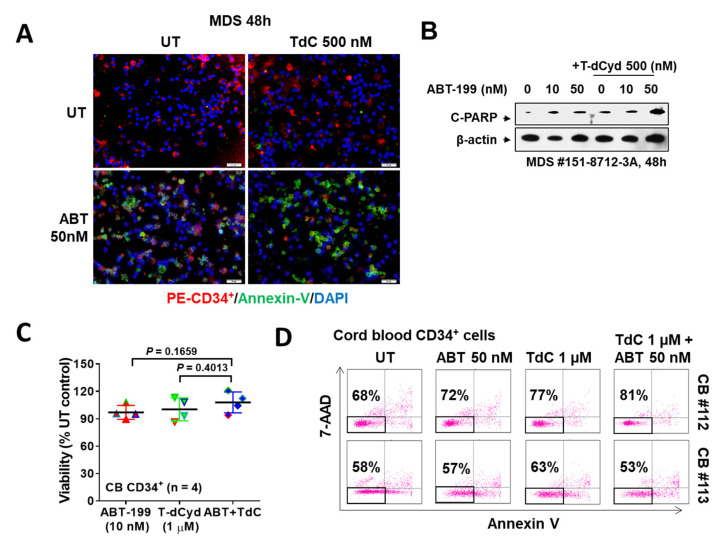
Bone marrow cells from an MDS patient (MDS-EB-1) were exposed (24 h) to 500 nM T-dCyd with or without 50 nM venetoclax, after which they were stained with PE-CD34^+^ (red) and Annexin (green) Abs. Cells were then viewed under an immunofluorescence microscope at 200× magnification (**A**). Cleaved PARP was detected by WB (**B**). Cord blood normal CD34^+^ cells (*n* = 4) were exposed (24 h) to 1 uM T-dCyd with or without 50 nM venetoclax, after which the CD34^+^ gated population was monitored for cell viability using Annexin V and 7-AAD staining (**C**). The percentage of viable cells in control and treated populations is reflected in the dot plot (**D**).

Bone marrow mononuclear cells from a patient with MDS were exposed to ABT-199 (50 nM) ± 500 nM T-dCyd for 48 h, after which apoptosis was monitored by Annexin-V/DAPI staining. Immunofluorescence revealed that while T-dCyd alone had no effect, and ABT-199 alone modestly increased apoptotic cells, the combined exposure resulted in a pronounced increase in apoptosis (Figure 3A). The Western blot analysis showed that the combined treatment sharply increased the rate of PARP cleavage (Figure 3B). In contrast, combined exposure to 10 nM ABT-199 and a higher concentration of T-dCyd (1 μM) exerted no significant effects on the viability of normal CD34^+^ cells (Figure 3C,D). These findings raise the possibility that the T-dCyd/ABT-199 regimen may selectively target primary MDS cells.

### 3.5. The T-dCyd/ABT-199 Regimen Increases Mitochondrial ROS Production in MDS-Derived Cell Lines

**Figure 4:**
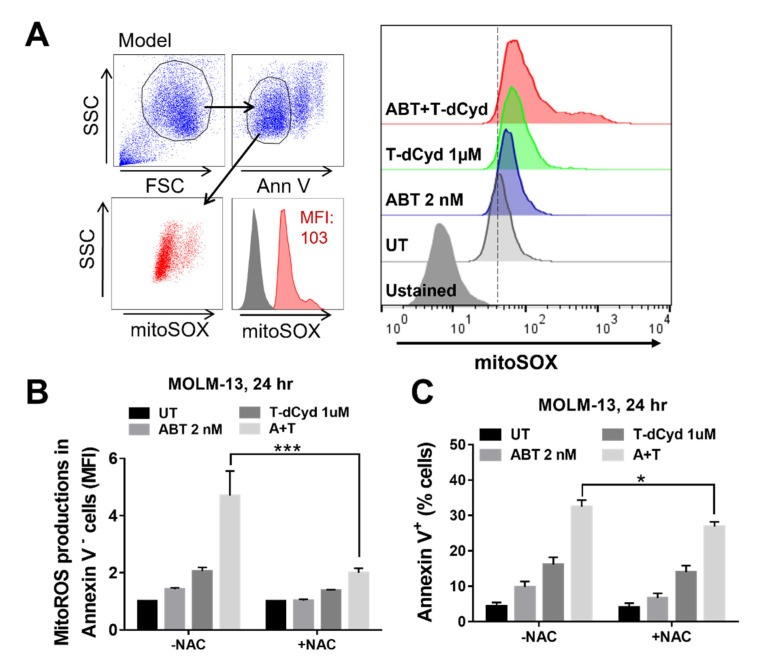
MOLM–13 cells were exposed (24 h) to the designated concentrations of ABT and T–dCyd, after which mitochondrial ROS in the viable cell population was monitored by FCM and MFI (**A**). (**B**) Effects of NAC on mitoROS (**B**) and viability (**C**) following the same exposure. For *p* values, * = <0.05; *** = <0.001.

A flow cytometric assay was employed to assess the effects of the regimen on mitochondrial ROS production based on comparison of mean fluorescence intensities (MFI) between the various treatment groups. Whereas ABT-199 and T-dCyd alone modestly increased the rate of mitochondrial ROS production in MOLM-13 cells, the combined exposure led to a clear increase, which manifested has a rightward shift in the histogram (Figure 4A). The quantification of the ROS levels showed a significant increase with the combined treatment compared to that of the single-agent exposure (Figure 4B). Very similar results were obtained in SKM-1 cells (Supplementary Figure S2). Moreover, in the MOLM-13 cells, the increase in ROS generation was abrogated by the co-administration of the free radical scavenger N-acetyl cysteine (Figure 4B). Significantly, the lethal effects of the ABT-199/T-dCyd regimen was partially, but significantly, reduced by the NAC co-administration (Figure 4C). Together, these findings raise the possibility that increased mitochondrial ROS production may contribute functionally to the lethal effects of the ABT-199/T-dCyd regimen in MDS-derived cells.

### 3.6. Combined Exposure of SKM-1 Cells to T-dCyd and ABT-199 Results in Diminished Expression of Nuclear Anti-Oxidant Proteins and BCL-2

**Figure 5:**
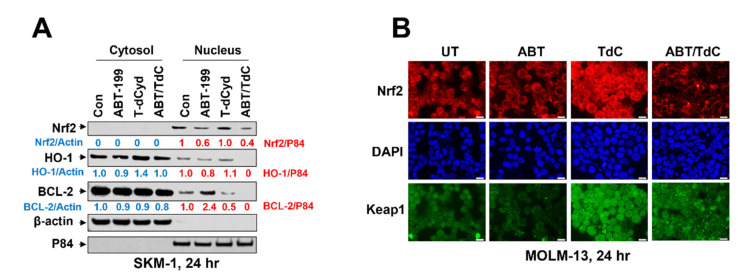
(**A**) SKM-1 cells were exposed (24 h) to ABT-199 ± T-dCyd (1 μM each), after which cytosolic and nuclear fractions were monitored for NRF2, HO-1, and BCL-2 expressions by WB analysis. Images were quantified and analyzed by using ImageJ software. Data were normalized to the ratio of the indicated protein and β-actin/P84 versus control. (**B**) MOLM-13 cells were exposed (24 h) to T-dCyd (500 nM) ± 2 nM ABT-199, after which the nuclear disposition of NRF2 was monitored by immunofluorescence microscopy.

The Western blot analysis of SKM-1 cells exposed to ABT-199 (1 µM) ± 1 µM T-dCyd for 24 h revealed a modest, but discernible, reduction of the nuclear expression of the anti-oxidant protein Nrf2 (Figure 5A). In addition, more pronounced reductions of the nuclear levels of another anti-oxidant protein, HO-1, along with BCL-2, were observed (Figure 5A). Notably, the nuclear levels of BCL-2 were increased with ABT-199 exposure, but this effect was reversed by the T-dCyd administration. In addition, the nuclear staining of Nrf2 and the anti-oxidant protein Keap1 [14] increased with T-dCyd exposure, but this effect was clearly reduced with the combined T-dCyd/ABT-199 treatment (Figure 5B). These findings raise the possibility that the diminished expression of various anti-oxidant proteins, and the resulting increase in the ROS levels, may contribute to the lethal effects of a combined T-dCyd/ABT-199 treatment in MDS cells.

## 4. Discussion

The results of this study indicate that co-administration of novel, orally available thio-deoxycytidine derivatives and hypomethylating agents (e.g., T-dCyd) interact synergistically with venetoclax (ABT-199) to induce apoptosis in the malignant myeloid cells of AML, as well as in their myelodysplastic (MDS) counterparts. The concept of combining ABT-199 with HMAs has been extensively explored in AML models, and initial studies have suggested that this interaction may reflect perturbations in mitochondrial function, particularly the disruption of mitochondrial Complex IV [5]. Furthermore, this strategy may be particularly effective in eradicating more primitive leukemic progenitors exhibiting stem cell-like characteristics [15]. Although there is considerably fewer data involving MDS models, one study demonstrated that the ABT-199/HMA strategy selectively targeted refractory MDS cells, while sparing normal hematopoiesis [16]. Notably, the combination of venetoclax and 5-azacytidine was effective against cells obtained from MDS patients who had progressed after HMA therapy [16]. Another pre-clinical study demonstrated the potential of 5-azacytidine activity in myeloid malignancies including MDS by BH3-mimetics, although ABT-737 was more effective than ABT-199 was [17]. Consistent with these findings, combining ABT-199 with established HMAs such as 5-azacytidine or aza-deoxycytidine has been approved for the treatment of older AML patients who are not candidates for more aggressive therapy [18]. Significantly, this strategy has also shown to be promising in patients with MDS [19]. Moreover, a retrospective analysis of high-risk MDS patients revealed good responses to the ABT-199/5-azacytidine regimen [20]. A meta-analysis of patients with MDS treated with ABT-199/5-azacytidine confirmed these results [19]. Taken together, these along with the present findings raise the possibility that combining orally active HMAs such as T-dCyd with BCL-2 antagonists such as ABT-199 may have potential utility in the treatment of relapsed/refractory MDS.

The combination of T-dCyd or its analog aza-T-dCyd with ABT-199 synergistically increased apoptosis in a variety of MDS or MDS-derived AML cell lines. These interactions were associated with the robust down-regulation of DNMT1, but this was also accompanied by the down-regulation of BCL-2. While the mode of action of HMAs has classically been attributed to DNMT1 degradation [21], the mechanism by which this occurs has never been definitively defined. In this context, BH3-mimetics such as ABT-199 are believed to act by disrupting BCL-2 function and preventing this molecule from interfering with the function of pro-apoptotic sensitizer proteins [22,23]. In any case, it may be relevant that the administration of T-dCyd is associated with BCL-2 down-regulation, which may act cooperatively with the disruption of BCL-2 function to promote cell death. Furthermore, the finding that the down-regulation of BCL-2 in an inducible system significantly enhanced T-dCyd lethality presents a strong argument that targeting BCL-2 represents an important functional contributor to the activity of this strategy.

Several lines of evidence argue that oxidative injury plays a key role in regulating the activity of the T-dCyd/ABT-199 regimen in MDS cells. Notably, the combined treatment was associated with a significant increase in reactive oxygen species, and co-administration of a free radical scavenger (e.g., NAC) significantly reduced both ROS generation as well as cell death. Such findings strongly suggest that this strategy acts at least in part by increasing free radicals. They are also consistent with evidence that the HMA/ABT-199 regimen acts by disrupting the mitochondrial electron transport chain [4,24]. It is noteworthy that previous studies in AML models showed that this regimen was associated with the down-regulation of several anti-oxidant proteins, e.g., Nrf2 and HO-1 [5]. The present results suggest that a similar mechanism may be operative in MDS cell models. Finally, it is important to note that early studies have demonstrated that disruption of BCL-2 function was associated with BID cleavage, ROS generation, and resulting cell death [25]. Thus, it is possible that T-dCyd may cooperate with ABT-199 in MDS cells to promote ROS production and Complex IV disruption, while down-regulating various anti-oxidant proteins, e.g., Nrf2 and HO-1, thereby disabling anti-oxidant defenses and increasing DNA damage. The net effect of these events is a pronounced increase in MDS cell death.

Finally, it is important to note that similar interactions between T-dCyd and ABT-199 were observed in primary MDS-derived mononuclear cells, but they did not occur in normal CD34^+^ cord blood cells. The basis for this in vitro selectivity remains to be determined, but it might reflect a reduced dependence of normal cells on BCL-2 for survival [11] and/or diminished responses of normal cells to the HMAs. Given the entry of HMAs such as T-dCyd into the clinical arena (NCT 02423057), the availability of an all-oral regimen incorporating such agents in combination with ABT-199 for patients with MDS could be an attractive possibility. Efforts to support such a strategy, e.g., through the performance of in vivo studies therefore appear to be justified.

## 5. Conclusions

A regimen combining the novel orally active HMA T-dCyd with ABT-199 kills MDS cells through an ROS-dependent mechanism, and this strategy warrants consideration for the treatment of MDS.

## Data Availability

All original source data (chiefly Western blot data) linked to the figures in the manuscript are available at the website OSFHOME. https://osf.io/c9nzt/?view_only=298b25f1907b4937b79a5fb4297c79fb (accessed on 10 January 2023).

## Supplementary Materials

The following supporting information can be downloaded at: https://www.mdpi.com/article/10.3390/hematolrep15010010/s1. Figure S1: ABT-199/T-dCyd or aza-T-dCyd synergism in AML cell lines; Figure S2: The T-dCyd/ABT-199 regimen increases mitochondrial ROS production in SKM-1 cells.
