# Supplementary material for: Synergistic Interactions between the Hypomethylating Agent Thio-Deoxycytidine and Venetoclax in Myelodysplastic Syndrome Cells
Source: Hematol Rep. 2023 Feb 2;15(1):91–100. doi: 10.3390/hematolrep15010010 (PMC9944092; doi:10.3390/hematolrep15010010)
Supplement: Supplementary file 1 [file hematolrep-15-00010-s001.zip › hematolrep-1873528-supplementary.pdf]

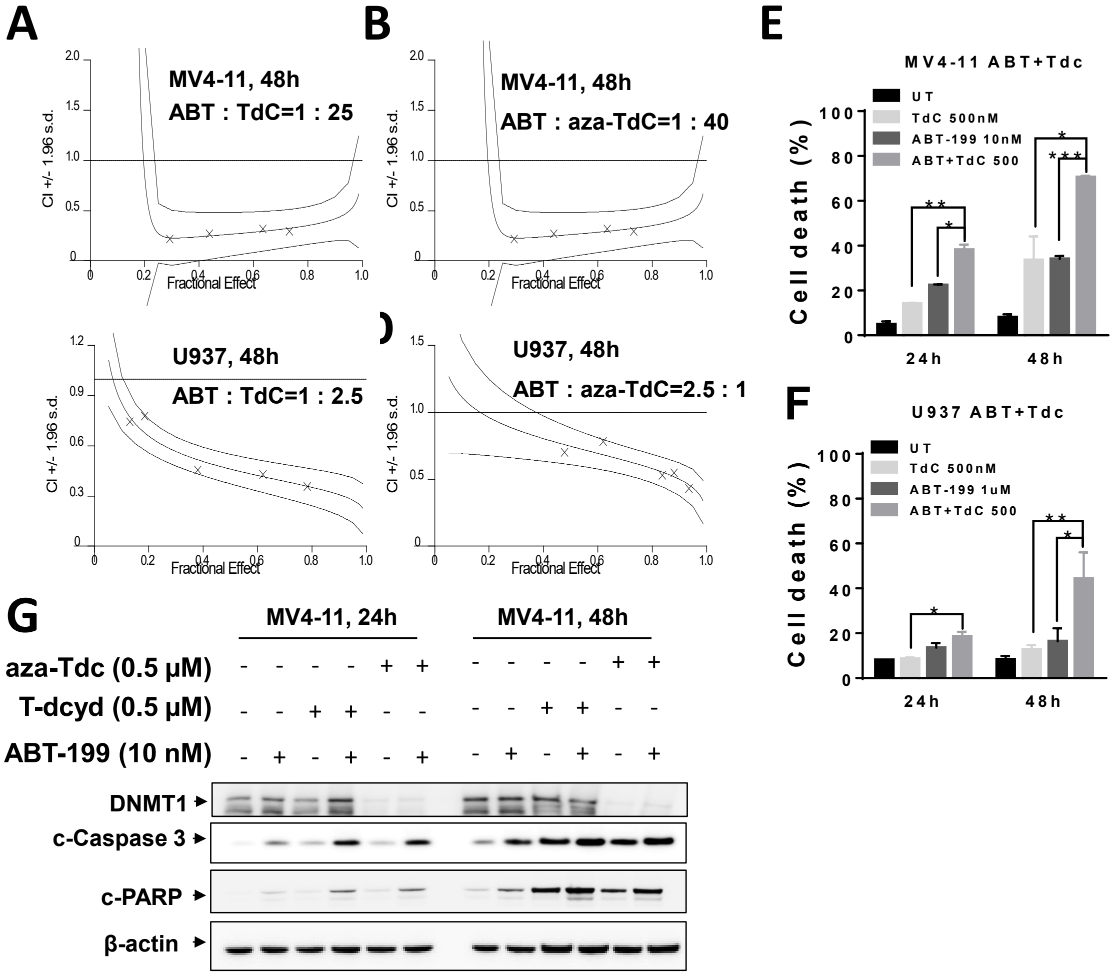


**Supplementary Figure S1.** MV4-11 and U937 cells were exposed to varying concentrations of ABT-199 and T-dCyd or Aza-T-dCyd administered at a fixed ratio for 48 h, after which the Median Dose Effect analysis was used to characterize concentration index (CI) values in relation to the fraction affected (FA). CI values < 1.0 denote synergistic interactions (A–D). (E,F) Cell death was monitored by Annexin V/PI staining and FCM. For p values, * = <0.05; ** = <0.01; *** = <0.001. (G) Cleaved PARP, caspase 3, and DNMT1 were detected by WB in MV4-11 cells exposed to T-dCyd ± ABT-199; β-actin controls are shown to document equivalent loading and transfer.

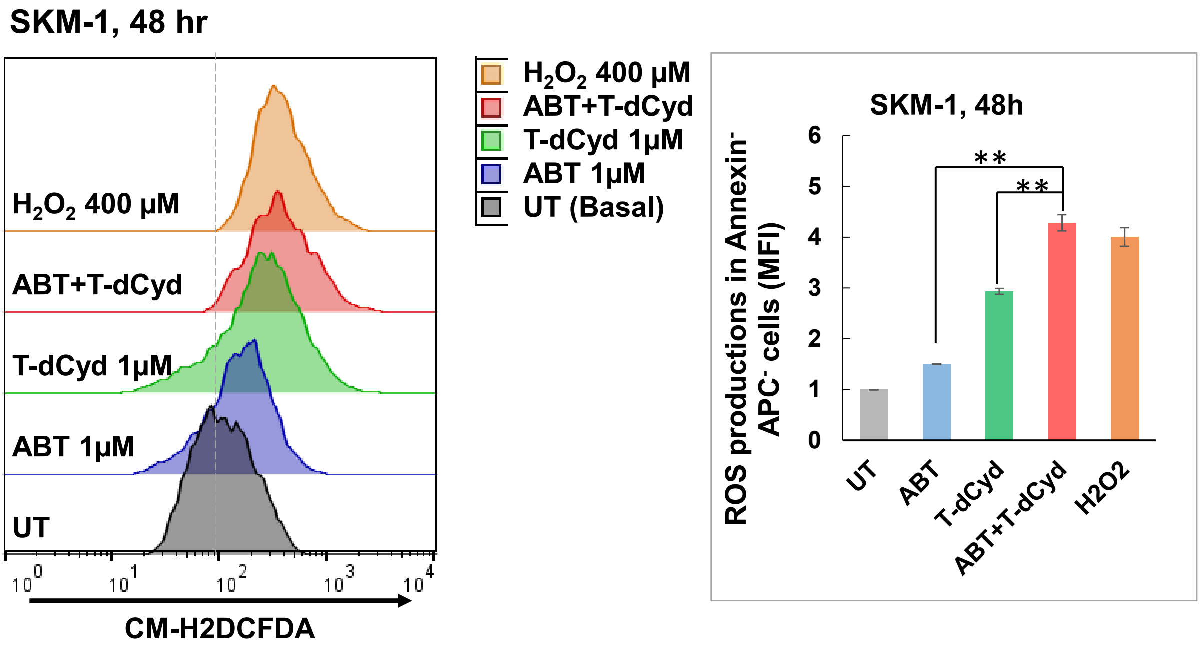


The ROS level of Annexin- / APC - (alive) cells

**Supplementary Figure S2.** SKM-1 cells were exposed (48 hr) to the designated concentrations of ABT and T-dCyd, after which mitochondrial ROS in the viable cell population was monitored by FCM and MFI. ** = < 0.01.
